# Interpersonal Psychotherapy as a Single Treatment for Borderline Personality Disorder: A Pilot Randomized-Controlled Study

**DOI:** 10.3389/fpsyt.2020.578910

**Published:** 2020-09-11

**Authors:** Paola Bozzatello, Silvio Bellino

**Affiliations:** Department of Neuroscience, University of Turin, Turin, Italy

**Keywords:** interpersonal psychotherapy (IPT), borderline personality disorder (BPD), waiting list, controlled trial, time-limited psychotherapy

## Abstract

Recent guidelines share the recommendations that psychotherapy plays a central role in the treatment of borderline personality disorder (BPD). In recent years, interpersonal psychotherapy adapted for treating BPD (IPT-BPD) was added to specific psychotherapies and was tested in combination with pharmacotherapy. The present study is aimed to assess the efficacy of IPT-BPD revised (IPT-BPD-R) as single treatment in a sample of BPD patients. Results obtained in a group of patients receiving IPT-BPD-R were compared with those observed in a control group in waiting list plus clinical management (WL/CM). Forty-three BPD subjects were randomly allocated to one of the two arms. Patients were assessed at baseline and after 10 months with the following assessment instruments: Clinical Global Impression Scale, Severity item (CGI-S), Social Occupational Functioning Assessment Scale (SOFAS), Borderline Personality Disorder Severity Index (BPDSI), Barratt Impulsiveness Scale, version 11 (BIS-11), Modified Overt Aggression Scale (MOAS), and Self Harm Inventory (SHI). Statistical analysis was performed with one-way analysis of variance (ANOVA) or chi-square test to compare baseline characteristics of the two treatment groups. Comparison of score changes at the end of the trial between the two groups was calculated for each rating scale with the analysis of variance for repeated measures. Seven patients (16.3%) discontinued the treatment in the first month of the trial for non-adherence. We found a significant effect within subjects (trial duration) for all rating scales, except for the MOAS. A significant effect between subjects (treatment modality) was found for CGI-S; SOFAS; BIS-11 total score and subscale “non-planning impulsivity”; BPDSI total score and items “interpersonal relationships,” “impulsivity,” and “identity.” So, results showed differences between groups in favor of psychotherapy in terms of reduction of severity of general psychopathology, improvement of social and occupational functioning, and decrease of global BPD symptoms and three specific domains. On the other hand, we did not find any differences between groups for self-harm behaviors and aggressive behaviors.

## Introduction

Borderline personality disorder (BPD) is a heterogeneous and severe psychiatric disorder, characterized by a pervasive instability in affects, impulse, sense of self, and interpersonal relationships. BPD reaches a point prevalence rate of 2.7% (1) and represents the most common personality disorder in clinical populations (2, 3). Because of the complexity of the disorder several sets of guidelines for the treatment of BPD were published in the last two decades, but no medication has obtained an official indication for it, yet. Recent guidelines from the National Institute for Health and Clinical Excellence of England and Wales (NICE) (4, 5) and the National Health and Medical Research Council (NHMRC) of the Australian Government (6) concluded that drug therapy was not recommended other than for treatment of other mental disorders in comorbidity or to control, during a crisis or a limited period, specific acute symptoms (7). Systematic reviews, meta-analyses, and guidelines share the opinion that psychotherapy plays a central role in BPD treatment (8–10). Several models of psychotherapy specifically developed for this disorder have been tested in single or combined trials (11–13). Among the most notable interventions are dialectical behavior therapy (DBT) (14) mentalization-based therapy (MBT) (15), and transference focused therapy (TFP) (16). In recent years, interpersonal psychotherapy was adapted for treating BPD patients (IPT-BPD) and was presented as an addition to the available therapeutic tools (17–22). IPT adapted for BPD derived from the standard model of IPT for major depression (23) modified by Markowitz (17) to address the peculiar features of BPD and to handle the difficulties in interpersonal relationships typical of these patients. Our research group proposed a revision of the IPT-BPD that is indicated as IPT-BPD-R (24).

With the purpose to begin studying the efficacy of this new proposal of psychotherapy we performed initial trials in samples of BPD patients who received a combination of IPT-BPD with medications commonly used in this clinical population (19–22). Results showed that combined treatment with IPT-BPD and fluoxetine was superior to single fluoxetine to treat three BPD core symptoms: disturbed interpersonal relationships, affective instability, and impulsive dyscontrol. It was also more effective to decrease severity of anxiety symptoms and to improve the subjective perception of quality of life (19). The increased effects of combined therapy on impulsivity, interpersonal dysfunctions, and quality of life were maintained during 2 years of follow-up (22).

The present pilot study is the first trial that was designed to assess the efficacy of our revision of IPT-BPD as single treatment of a sample of BPD patients. Results obtained in patients receiving IPT-BPD-R were compared with those observed in a control group of patients in waiting list plus clinical management (WL/CM).

## Materials and Methods

### Participants

Forty-three consecutive outpatients with a diagnosis of BPD according with Diagnostic and Statistical Manual of Mental Disorders, 5th Edition (DSM-5) criteria (25) were recruited for this study. All participants were aged between 18 and 60 years. Patients attended the Center for Personality Disorders of the Department of Neuroscience at the University of Turin, Italy. The psychiatric diagnosis was made by an expert clinician and was confirmed with the Structured Clinical Interview for DSM 5 Clinical Version and Personality Disorders (26, 27). Exclusion criteria were: 1) a diagnosis of dementia or other cognitive disorders, schizophrenia or other psychotic disorders, or bipolar disorders, 2) a co-occurring major depressive episode and/or substance abuse, and 3) the administration of psychotropic medications and/or psychotherapy in the 3 months preceding the beginning of the study. Female patients of childbearing age were excluded if they were not using adequate birth control methods. This exclusion criterion was needed as patients in this sample were also evaluated before and after treatment with functional magnetic resonance. Patients were re-assessed at the end of the study to investigate if they still fulfilled DSM-5 diagnostic criteria for BPD. Assessment was made considering a time period of 1 month.

The group of 43 BPD subjects was randomly allocated to: 1) IPT-BPD-R (N = 22 patients) or 2) WL/CM (N = 21 patients). Research Randomizer (Urbaniak and Plous, Social Psychology Network Wesleyan University, Middletown, CT), a free web-based service for randomization, was used.

The 22 patients who received IPT-BPD-R and the 21 patients who were in WL/CM are matched for gender, age, and education (number of years completed at school and university referred by patients and confirmed by school and academic certificates). Psychotherapy was provided by therapists who were certificated by the Italian Society for Interpersonal Psychotherapy according to the international guidelines for IPT training and had at least 5 years of experience practicing IPT-BPD. Sessions of psychotherapy were steadily supervised by a senior psychotherapist (S.B.) with particular attention to check for fidelity to the manual.

Each subject voluntarily participated in the study, after providing written informed consent. We observed the Declaration of Helsinki guidelines. Approval was obtained from the ethics committee of the University Hospital “Città della Salute e della Scienza—Ospedale dell’Ordine Mauriziano” of Turin. The trial was registered as a part of a study of functional neuroimaging in the Australian New Zealand Clinical Trials Registry (ANZCTR) and allocated the code: ACTRN12619000078156.

### Measures

Patients were assessed at baseline and after 10 months with the following assessment instruments:

1. The Clinical Global Impression Scale, Severity item (CGI-S) (28),
2. The Social Occupational Functioning Assessment Scale (SOFAS) (29),
3. The Borderline Personality Disorder Severity Index (BPDSI) (30),
4. The Barratt Impulsiveness Scale, version 11 (BIS-11) (31),
5. The Modified Overt Aggression Scale (MOAS) (32),
6. The Self Harm Inventory (SHI) (33).

The CGI is a clinician-rated instrument to make global assessment of illness and consists of three different measures: severity of illness, global improvement, and efficacy index. In this study we considered severity of illness. It is a seven-point scale ranging from 1 (normal) to 7 (extremely ill).

The BPDSI is a semi-structured clinical interview assessing frequency and severity of BPD related symptoms. The interview consists of eight items scored on a 10-point frequency scale (0 = never; 10 = daily), including “abandonment,” “inter-personal relationships,” “impulsivity,” “para-suicidal behavior,” “affective instability,” “emptiness,” “outbursts of anger,” “dissociation and paranoid ideation,” and of one item scored on a four-point severity scale, concerning “identity.” The BPDSI showed adequate reliability and construct validity also in the Italian version (34).

The BIS-11 is a 30-items self-report questionnaire measuring the trait of impulsivity on a 4-point Likert scale. Higher scores for each item indicate higher levels of impulsivity. Twelve items are reverse-scored, in order to avoid response sets. Is it possible to identify three factors: cognitive impulsivity, motor impulsivity, and non-planning impulsivity. Global score is obtained by the sum of these factors. The BIS-11 showed adequate reliability and construct validity in both USA (35) and Italian (36) samples.

The MOAS is a clinician-rated scale consisting of four subscales for different types of aggression (verbal aggression, aggression against objects, aggression against others, and self-aggression). The subscales are rated on a 5-point scale (score 0–4). Higher scores for each subscale reflect the higher severity of a subject’s aggressiveness (32, 37).

The SHI is a brief, self-reporting instrument that provides informative data about clinically-relevant self-harm behaviors. Although not considered in the scoring, many of the SHI items provide additional information regarding the number of times a patient engages in a self-destructive act, as well as how recently he or she has done so. The scoring of the instrument is easily determined by counting the number of self-harm behaviors (38).

Assessment was performed by an investigator (P.B.) who received training session on psychometric instruments prior to start investigation.

At the end of the trial we also assessed how many patients still fulfilled diagnostic criteria for BPD. Clinical assessment was performed and Structured Clinical Interview for DSM-5 Disorders (SCID-5)-PD was re-administered.

### Treatment Conditions

#### Interpersonal Psychotherapy Adapted for Borderline Personality Disorder—Revised

IPT was initially developed by Klerman in 1984 (23) to treat major depression, but some investigators have successfully extended its indications to other psychiatric disorders (17, 39–45). Markowitz (17, 18) proposed an adaptation of IPT to the treatment of patients with BPD. IPT-BPD departs from the standard acute treatment of major depression to suit the needs of this particular and severe population of patients. Features of adaptation involve: different conceptualization of the disorder (BPD is defined as a mood-inflicted chronic illness similar to dysthymic disorder but with sporadic outbursts of anger); prolonged length of treatment (a first acute phase of 18 IPT sessions and a continuation phase of 16 sessions, up to 34 IPT sessions over 8 months); and flexibility of setting (a 10-min telephone contact once a week is provided) to handle crises and minimize the risk of therapeutic ruptures. In the last years, we designed a proposal of revision (IPT-BPD-R) of Markowitz’s adaptation of IPT to BPD in the attempt to overcome some limitations of this model that emerged during clinical practice. IPT-BPD-R consists of 10 months of therapy divided into two phases of 22 sessions (20 weeks) and 20 sessions (20 weeks). Sessions duration is of 50 min. Three additional sessions can be provided if patients present serious difficulties during the termination phase. Two weekly contacts by phone with the therapist are allowed in situations of crisis, as well as two admissions to hospital for a brief period of 7–10 days. During the hospitalization IPT-BPD-R continues if the patient’s clinical conditions allow it. In our revised model of IPT-BPD is also included an intervention for patients’ family members in order to help them to understand and deal with the disorder of their relative (24).

### Waiting List and Clinical Management

BPD patients who did not receive psychotherapy were monitored with 50-min clinical visits (assessment of symptoms and supportive intervention) with a frequency of once monthly. No medications were administered in this group.

### Statistics

Statistical analysis was performed with one-way analysis of variance (ANOVA) or chi-square test to compare baseline characteristics of the two treatment groups (age, gender, level of education, and clinical and functional rating scales scores). Comparison of score changes at the end of the trial between the two groups was calculated for each rating scale with the analysis of variance for repeated measures.

The software IBM SPSS Statistics version 26 (IBM Corp, 1989, 2019) was used for the analyses.

*P* values were considered significant when ≤ 0.05.

## Results

Forty-three patients were randomly assigned to 1) IPT-BPD-R (N=22) or to 2) WL/CM (N=21). Thirty-six out of the 43 patients (83.7%) completed the 10 months of the trial: 19 patients (52.7% of the completers) received IPT-BPD-R, while 17 patients (47.2%) was in WL/CM. Seven patients of the recruited sample (16.3%) discontinued the treatment in the first month of the trial for non-adherence to study protocol: three in the group who received IPT-BPD-R and four in the group in WL/CM. The final sample resulted in 36 patients with the mean age of 35.42 ± 14.71 years; they were 13 females (68.4%) and 6 males in IPT-BPD-R group and 11 females (64.7%) and 6 males in WL/CM group. No differences between the two groups were found concerning age, gender, and level of education with the ANOVA and chi square test.

Results of the ANOVA calculated for baseline mean scores of rating scales are reported in **Tables 1** and **2**. No significant differences between groups were found at baseline between the two treatment arms.

**Table 1:** Comparison of the baseline values of symptoms and functioning rating scales between the Interpersonal Psychotherapy Adapted to Borderline Personality Disorder-Revised (IPT-BPD-R) and WL/CM groups.

Scale	IPT-BPD-R (N=22)Mean ± SD	WL/CM (N=21)Mean ± SD	ANOVA*P*
CGI-S	4.00 ± 0.82	4.20 ± 0.79	0.584
BIS-11 total score	80.22 ± 9.30	81.13 ± 5.96	0.337
BIS-11 non-planning	26.41 ± 4.53	26.72 ± 5.01	0.682
BIS-11 motor	30.74 ± 6.23	31.04 ± 6.11	0.682
BIS-11 attentive	22.57 ± 5.44	21. 87± 6.75	0.263
MOAS	4.50 ± 2.61	4.20 ± 3.11	0.620
SHI	7.80 ± 4.02	7.33 ± 2.27	0.454
SOFAS	57.10 ± 7.06	55.50 ± 9.27	0.167

SD, standard deviation; ANOVA, analysis of variance; CGI-S, Clinical Global Impression scale—Severity item; SOFAS, Social Occupational Functioning Assessment Scale; BIS-11, Barrett Impulsiveness Scale—version 11; MOAS, Modified Overt Aggression Scale; SHI, Self Harm Inventory; IPT-BPD-R, Interpersonal Psychotherapy Adapted to Borderline Personality Disorder-Revised; WL/CM, waiting list plus clinical management.

**Table 2:** Comparison of baseline values of Borderline Personality Disorder Severity Index (BPDSI) total score and single items between the Interpersonal Psychotherapy Adapted to Borderline Personality Disorder-Revised (IPT-BPD-R) and waiting list plus clinical management (WL/CM) groups.

Scale	IPT-BPD-R (N=22)Mean ± SD	WL/CM (N=21)Mean ± SD	ANOVA *P*
BPDSI total score	48.09 ± 5.85	47.45 ± 6.32	0.630
Abandonment	7.32 ± 1.15	7.54 ± 1.32	0.321
Interpersonal relationships	8.01 ± 0.27	7.31 ± 1.82	0.413
Identity	2.28 ± 1.14	2.31 ± 1.29	0.403
Impulsivity	7.12 ± 1.31	6.92 ± 1.73	0.332
Parasuicidal behaviors	1.30 ± 1.50	1.42 ± 1.30	0.237
Affective instability	7.52 ± 0.89	6.84 ± 1.05	0.276
Outbursts of anger	6.30 ± 1.55	6.88 ± 1.43	0.327
Emptiness	5.60 ± 1.05	4.82 ± 1.34	0.182
Dissociation/paranoid ideation	2.38 ± 1.89	2.59 ± 1.82	0.562

SD, standard deviation; ANOVA, analysis of variance; BPDSI, Borderline Personality Disorder Severity Index; IPT-BPD-R, Interpersonal Psychotherapy Adapted to Borderline Personality Disorder-Revised; WL/CM, waiting list plus clinical management.

Results of the ANOVA for repeated measures to evaluate the effect of trial duration (within-subjects effect) and of treatment modality (between-subjects effect) on the score changes after 10 months are reported in **Table 3**.

**Table 3:** Significant results of the ANOVA for repeated measures calculated in the completers to compare the changes of the Clinical Global Impression Scale, Severity item (CGI-S), Social Occupational Functioning Assessment Scale (SOFAS), Barratt Impulsiveness Scale, version 11 (BIS-11) total score and “non-planning impulsivity” subscale, Borderline Personality Disorder Severity Index (BPDSI) total score and items “impulsivity,” “interpersonal relationships,” and “identity diffusion” scores, and Self Harm Inventory (SHI) score between treatment groups.

Scale	Treatment	BaselineMean ± SD	10 monthsMean ± SD	Within-subjects effect (duration)	Between-subjects effect (treatment)
CGI-S	IPT-BPD-R WL/CM	4.00 ± 0.82 4.20 ± 0.79	3.10 ± 0.93 4.08 ± 0.74	P=0.032	P=0.009
SOFAS	IPT-BPD-R WL/CM	57.10 ± 7.06 55.50 ± 9.27	68.22 ± 8.08 57.10 ± 8.73	P=0.028	P=0.02
BIS-11 total score	IPT-BPD-R WL/CM	80.22 ± 9.30 81.13 ± 5.96	64.78 ± 12.74 77.37 ± 5.51	P=0.001	P=0.031
BIS-11 non-planning	IPT-BPD-R WL/CM	26.41 ± 4.53 26.72 ± 5.01	20.82 ± 4.61 25.18 ± 5.12	P=0.001	P=0.042
BPDSI total score	IPT-BPD-R WL/CM	48.09 ± 5.85 47.45 ± 6.32	36.09 ± 8.57 44.57 ± 6.53	P=0.001	P=0.01
Interpersonal relationships	IPT-BPD-R WL/CM	8.01 ± 0.27 7.31 ± 1.82	4.35 ± 1.6 6.79 ± 1.71	P=0.001	P=0.001
Identity disturbance	IPT-BPD-R WL/CM	2.28 ± 1.14 2.31 ± 1.29	1.58 ± 1.23 2.29 ± 1.07	P=0.001	P=0.014
Impulsivity	IPT-BPD-R WL/CM	7.12 ± 1.31 6.92 ± 1.73	4.01 ± 1.18 6.43 ± 1.47	P=0.001	P=0.03
SHI	IPT-BPD-R WL/CM	7.80 ± 4.021 5.03 ± 2.27	6.91 ± 5.12 4.12 ± 3.91	P=0.005	P=0.271

SD, standard deviation; ANOVA, analysis of variance; CGI-S, Clinical Global Impression scale—Severity item; SOFAS, Social Occupational Functioning Assessment Scale; BIS-11, Barrett Impulsiveness Scale—version 11; BPDSI, Borderline Personality Disorder Severity Index; SHI, Self Harm Inventory; IPT-BPD-R, Interpersonal Psychotherapy Adapted to Borderline Personality Disorder-Revised; WL/CM, waiting List plus clinical management.

We found a significant effect within subjects (trial duration) for all rating scales (*P* values ranged between 0.001 and 0.032), except for the MOAS (*P* = 0.12).

A significant effect between subjects (treatment modality) was found for CGI-S (*P* = 0.009); for SOFAS (*P* = 0.02); for BIS-11 total score (*P* = 0.031) and subscale “non-planning impulsivity” (*P* = 0.042); for BPDSI total score (*P* = 0.01) and items “interpersonal relationships” (*P* = 0.001), “impulsivity” (*P* = 0.03), “identity” (*P* = 0.014).

DSM-5 diagnostic criteria for BPD were still met by the 36 patients who completed the trial.

## Discussion

The present randomized controlled trial is to our knowledge the first study that specifically investigated the efficacy of interpersonal psychotherapy adapted for borderline personality disorder as single therapy in comparison with waiting list and clinical management in a group of moderately ill patients with a DSM-5 diagnosis of BPD. Previous studies found that IPT may be considered a useful treatment tool for these patients, who show prominent relational problems, but the majority of data derived from trials that used IPT-BPD in combination with medications (18–22).

All subjects in this sample still fulfilled DSM-5 diagnostic criteria for BPD at the end of the trial. A possible reason of this finding is that a time-limited psychotherapy has a significant effect in decreasing severity of BPD symptoms and improving global functioning, but it does not produce such a change of personality organization to obtain a full remission of the disorder. We assessed the diagnosis of BPD at the end of trial considering a time period of 1 month. In our opinion, this period is appropriate to measure changes after a time-limited psychotherapy, in accordance with previous studies of short-term therapies of BPD (46).

An interesting result of this study is that both groups of patients and controls obtained a significant improvement in all outcome measures after 10 months of observation, except for the measure of aggressive behaviors that did not significantly change in the two arms. This finding suggested that in BPD taking charge of the patient may contribute by itself to induce a clinical and functional improvement, independently by the specificity of the therapy. Concerning aggressive behaviors, in this study we found that neither interventions (IPT and WL/CM) obtained a significant improvement. A possible explanation is that in our sample MOAS scores at baseline were rather low (with a mean lower than 5).

Results of the ANOVA also showed differences between groups in favor of psychotherapy in terms of reduction of severity of general psychopathology, improvement of social and occupational functioning, and decrease of global BPD symptoms and specific domains. In particular, patients who received IPT-BPD-R presented a greater improvement on impulsive behavioral dyscontrol, interpersonal disturbance, and identity diffusion in comparison with patients in WL/CM. On the other hand, we did not find any differences between groups on self-harm behaviors and aggressive behaviors.

In previous investigations of our research group, we observed that the addition of IPT-BPD to medication produced greater effects on some BPD core symptoms, as impulsive dyscontrol and interpersonal relationships disturbance with advantages that endured during a follow-up period of 2 years (19, 22). In the present study these results were confirmed in patients who received IPT-BPD-R as single treatment. So, we can suppose that the improvement of impulsive behaviors and interpersonal relationships in patients depend on the specific effect of interpersonal psychotherapy. In fact, IPT-BPD is a model of psychotherapy that is aimed to improve BPD psychopathology by a modification of dysfunctional interpersonal patterns. Better abilities to organize interpersonal relationships obtained with this therapy are expected to promote a more stable social environment with consequently a more successful control of impulsive reactions.

When we compared our results with those drawn by studies that evaluated the efficacy of other models of psychotherapy, such as DBT, MBT, and TFP, we found some overlapping effects, but also differences among the four treatment models. For example, the greater effect of IPT *versus* WL/CM on general psychopathology and global functioning found in the present study is shared by DBT (47, 48), MBT (49), and TFP (50, 51) in comparison with treatment as usual or general clinical management. It is not particularly surprising that general psychiatric symptoms and functioning improve with psychotherapy independently of the specific design of intervention, but it is noticeable that these improvements derived by specific psychotherapies rather than general supportive treatments.

Considering the core BPD symptoms, a result that is particularly worthy of attention concerns the effect of IPT-BPD-R in regulating self- and hetero- evaluated impulsivity. We found that non-planning impulsivity, typical of BPD subjects, is particularly sensitive to the IPT intervention. As far as we know, no other studies on the efficacy of psychotherapy in BPD obtained the same finding, except for our previous study of IPT-BPD combined with medication and a single trial that indicated a small effect of DBT on impulsivity in BPD patients (52). So, we can hypothesize that the effect of IPT-BPD on impulsive symptoms is rather specific for this model of psychotherapy.

On the contrary, several trials conducted on DBT registered a significant reduction of self-harm behaviors that was not observed in our sample. We can suggest that DBT is specific in reducing self-aggressive behaviors that commonly occur in patients with BPD (8, 14, 48, 52, 53).

In our investigation, disturbance of interpersonal relationships showed a better improvement in patients treated with IPT-BPD-R compared with controls. Similar findings were reported in two studies performed on the efficacy of MBT in partially-hospitalized BPD patients (15) and in BPD outpatients (49). The common effect of IPT and MBT in restoring interpersonal relationships of BPD patients can be due to the fact that both psychotherapies, with different techniques, are aimed to ameliorate the reflective function of these patients and their abilities to understand thoughts and emotions of others.

Another remarkable result of our trial concerns the effect of IPT-BPD-R on identity disturbance. We observed that patients who received IPT-BPD-R showed a greater reinforcement of identity integration than patients in WL/CM. In accordance with our finding, some authors observed that time-limited psychotherapy (in particular data concerned dialectical behavior psychotherapy) can improve identity disturbance in patients affected by BPD enhancing the patient’s sense of self and coherence in their feelings and thoughts (54–56).

The incapacity to maintain a stable and cohesive sense of self in BPD patients involves a precarious and troubled world view in the context of interpersonal interactions. So, a model of psychotherapy focused on interpersonal problems, like IPT, encourages the person to establish more balanced, realistic, and intimate relationships, resulting in a more coherent sense of self (21).

However, this result requires to be considered with caution and needs to be replicated to draw any reliable conclusions. In fact, we only found a significant score change of the item “identity disturbance” of the BPDSI, but we did not assess the level of identity integration with specific evaluation instruments.

The present study presented some points of strength, such as the randomized controlled design, and the use of both self-evaluated and clinician-rated instruments to obtain a more reliable assessment.

Moreover, patients who completed the trial were 83.7% of the initial sample, which can be considered a very good proportion in a sample of BPD patients.

On the other hand, our findings are affected by some limitations. The first limit concerns the relatively small size of the clinical sample. A second limitation is possibly due to the exclusion of psychiatric comorbidities. In fact, this choice has the relevant purpose to avoid the effects of coexisting psychiatric disorders on treatment response, but has the unfavorable consequence that clinical characteristics of our patients are partly different from those found in clinical practice and can limit generalizability of our findings. So, this is a relevant issue in choosing selection criteria of BPD patients for clinical trials. Another limit was that assessment instruments did not include a scale designed to evaluate the specific mechanisms of action of IPT-BPD.

In summary, our pilot trial is aimed to test the efficacy of IPT-BPD-R as single therapy of BPD patients. Subjects treated with this psychotherapy showed a greater improvement in general psychopathology, social and occupational functioning, and global and specific BPD symptoms (impulsive behavioral dyscontrol, interpersonal disturbance, and identity diffusion) in comparison with control patients in WL/CM.

On the basis of our data and those derived from studies that were performed to assess the efficacy of other psychotherapeutic interventions, we can conclude that the main psychotherapies available for BPD patients produce comparable effects on general psychopathology and global functioning, but different models seem to be targeted on specific symptoms domains. IPT-BPD-R appeared to induce a more specific effect on impulsive dyscontrol, interpersonal relationships, and identity disturbance. However, these are initial results that need to be replicated before considering their implementation in clinical practice.

## Data Availability Statement

The datasets presented in this article are not readily available because our Ethical Committee does not allow communication of data collected in our University Hospital. Requests to access the datasets should be directed to silvio.bellino@unito.it.

## Ethics Statement

The studies involving human participants were reviewed and approved by Ethics committee of the University Hospital “Città della Salute e della Scienza—Ospedale dell’Ordine Mauriziano” of Turin. The patients/participants provided their written informed consent to participate in this study.

## Author Contributions

Authors equally contributed to perform the study and prepare the article.

## Funding

Program of the Ministry of Health of the Republic of Italy to fund the Department of Excellence.

## Conflict of Interest

The authors declare that the research was conducted in the absence of any commercial or financial relationships that could be construed as a potential conflict of interest.

The reviewer FB declared a shared affiliation with the authors to the handling editor at time of review.
